# Quality assurance checklist and additional considerations for canine clinical genetic testing laboratories: a follow-up to the published standards and guidelines

**DOI:** 10.1007/s00439-019-02013-9

**Published:** 2019-04-13

**Authors:** Lisa G. Shaffer, Anja Geretschlaeger, Christina J. Ramirez, Blake C. Ballif, Casey Carl

**Affiliations:** 1 Paw Print Genetics, Genetic Veterinary Sciences, Inc., 220 E Rowan, Suite 220, Spokane, WA 99207 USA; 2 Feragen GmbH, Salzburg, Austria

## Abstract

There is currently no oversight for canine clinical genetic testing laboratories. We published an initial set of standards and guidelines with the goal of providing a basis for which canine testing laboratories could evaluate their quality assurance programs. To further those standards and guidelines, we have developed a checklist that can be used as a self-evaluation to identify gaps in their programs for continual quality improvement over time. Because there is currently no organization willing to oversee an external proficiency program, the checklist provides the first step toward an internal, self-assessment that can be used periodically to monitor improvements. In addition, we attempt to address concerns from the canine community regarding rare or private mutations, genetic screening using array-based technologies, non-peer reviewed tests that are being offered, and the clinical validity of certain mutations in particular breeds. Through coordination, conversation and hard work, the canine genetic testing community can strive to organize to improve testing and to provide more transparency to consumers and better outcomes for dogs.

**Electronic supplementary material:**

The online version of this article (10.1007/s00439-019-02013-9) contains supplementary material, which is available to authorized users.

## Introduction

The role and responsibility of the clinical laboratory are to translate new genetic discoveries into clinical tests to make them available to breeders and dog owners with the goal of improving breeding programs and the overall health of dogs. Providing quality testing for companion animals goes beyond just good laboratory practices. Information contained on websites must be presented in a way that the consumer can understand what they are ordering prior to testing. Genetic counseling should be available to the owner or breeder prior to ordering their testing and at the time that they receive their results. The results from the tests offered should have medically actionable outcomes, even if it is just simply to inform the breeder on how to pair dogs for breeding to avoid producing puppies with genetic diseases. To help laboratories in this mission, we published the first set of standards and guidelines for clinical canine genetic testing laboratories (Shaffer et al. 2018). To further the understanding of how these standards and guidelines can be used in the clinical laboratory and to address some concerns since its publication, we will take a ‘deeper dive’ into the world of clinical genetic testing and present a checklist that can be used by laboratories wishing to identify gaps in their current quality assurance programs with the goal of continual improvement.

This issue of *Human Genetics,* dedicated to canine genetics, provides many examples that demonstrate the advances made in canine genetic disease discovery and testing. At the time of this publication, more than 350 causal variants have been identified in the dog (Online Mendelian Inheritance in Animals 2019). For a given disease, canine mutations have been identified in novel genes, while others are found in the canine orthologue of the human gene. In either case, dogs can serve as a model for further study of the human condition. Once published, each mutation becomes available to be developed and validated as a clinical test. Breeders are increasingly becoming more reliant on these clinical tests to aid in their breeding decisions.

Some argue that it is the responsibility of the scientists who identify disease-associated genetic variants to determine which discoveries are appropriate to develop into commercially available DNA tests for dog breeders (Mellersh 2012). However, in many cases, the research laboratory in which the mutations are identified may not have the financial means or have access to samples, to test the mutation across large numbers of dogs within a breed and across breeds. In general, clinical laboratories are more likely to have access to such samples and can more accurately determine how widespread a mutation is in a breed or in dogs in general.

As more breeders use DNA testing to inform breeding decisions, concerns have been voiced regarding how these discoveries become clinical tests available to the public (Moses et al. 2018). The previous lack of oversight can now start to be addressed through the use of the published standards and guidelines (Shaffer et al. 2018). Through coordination, conversation and hard work, the canine genetic testing community can strive to organize, much like the American College of Medical Genetics and Genomics (ACMG) has done for human genetic testing over the past 30 years (Rimoin 2011), to improve genetic testing, breeder counseling and to produce better outcomes for dogs.

## Standards and guidelines for clinical canine genetic testing laboratories

The purpose of standards and guidelines is to provide uniformity and guidance toward quality improvement across laboratories in the industry. This is achieved through two factors. First, the individual efforts of each laboratory to implement protocols that help ensure high quality testing and accuracy, and second, an unbiased organization to help disseminate information and oversee the progress of the individual laboratory and the industry while maintaining the goals of continuous improvement over time and implementation of changes as technologies evolve and the genetic targets become more complex.

We published the first set of standards and guidelines for genetic testing in domestic dogs in the hopes of providing a baseline for testing standards and to bring together the clinical laboratories to continue to discuss and improve these standards to provide consistency and high accuracy across the entire industry (Shaffer et al. 2018). A recent opinion piece speculates that few laboratories would be able to comply with the minimal standards published (DogWellNet.com 2019). However, these standards and guidelines were based on those produced over the years by the ACMG with the canine recommendations limited in scope after examining them under the lens that genetic testing for dogs is still considered an emerging clinical discipline. Given this, we divided the current standards for human testing into minimal standards and desired standards, in the effort to provide a baseline standard that most canine clinical testing laboratories should be able to fulfill with a higher level, desired standard goal to achieve. If we set the standards so low to be inclusive of all laboratories, no matter their quality, it defeats the purpose of quality improvement and the willingness to strive for accuracy in testing.

## Who should be the providers of canine clinical testing?

Attempts by others have been made to distinguish commercial laboratories from university and research laboratories, trying to set them apart in their ability to have quality assurance programs (DogWellNet.com 2019). However, in human clinical genetic testing, all laboratories must meet the standards of the Clinical Laboratories Improvement Amendment of 1988 (CLIA’88) regulations (American College of Medical Genetics and Genomics Standards and guidelines for clinical genetic laboratories 2019) regardless of whether they are a commercial laboratory or based in a university. Because most research laboratories do not or cannot achieve CLIA’88 standards, human testing is restricted to commercial and university clinical laboratories. Research laboratories in human genetics cannot report results to patients and research studies are conducted only under institutional review board oversight. Because canine genetic testing laboratories do not fall under CLIA’88, it seems reasonable that testing laboratories should strive to achieve at least the minimal standards recently published for clinical diagnostic testing for dogs (Shaffer et al. 2018). Research laboratories performing only one or a few canine tests likely cannot meet the criteria presented in the standards and guidelines and although the researcher that discovered the mutation is clearly skilled in genetic methodologies, they likely do not have the quality assurance protocols and methods-based procedures (Schrijver et al. 2014) in place to minimize sample mix-up or contamination, nor the personnel to oversee such a program. Following the example set by human genetic clinical testing, canine genetic testing reported directly to the consumer or veterinarian should also be restricted to commercial and university laboratories that meet the minimal standards as outlined (Shaffer et al. 2018).

## Quality assurance checklist

To further the adoption of the standards and guidelines, we have developed a checklist that can be used for self-assessment (see supplemental materials). Again, taking experience from the ACMG and the College of American Pathologists, external proficiency involves performing surveys 2–3 times per year with laboratory challenges that are graded against a consensus of laboratories participating in that specific survey (Brothman et al. 2011; Feldman et al. 2014). In addition, there is an onsite inspection component and a self-assessment component. Although canine clinical genetics laboratories do not have an organized body currently willing to take on the task of external proficiency, the checklist provided can be used by the laboratories to perform self-evaluations to identify gaps in their own quality assurance programs (S1). The purpose of the checklist is to provide a way for the laboratory to establish a baseline and allow planning and implementation of improvements over time.

There are likely few canine genetic testing laboratories today that can check all the boxes on this self-assessment, but it represents a means for evaluating and measuring improvements in the areas outlined such as quality, personnel, consumer education and laboratory methods. No such document exists currently for canine genetic testing laboratories even though some laboratories were established well over 20 years ago. The fact that few canine clinical laboratories can check all the boxes on this self-assessment today should not be of concern. The reason for providing such a document is to allow laboratories to identify gaps and work toward improvement over time.

The standards and guidelines (Shaffer et al. 2018) and the checklist provided herein should not be considered static documents. Rather, they are dynamic, living documents that should be modified and amended from time to time as new technologies and clinical or testing issues arise within the discipline. Illustrative examples that have been developed by the ACMG include the use of microarray analysis in neoplasia (Cooley et al. 2013), interpretation of sequence variants (Richards et al. 2015), and laboratory analysis of organic acids (Gallagher et al. 2018).

## Transparency in information to consumers

Many breeders have become quite competent in interpreting their genetic results when using a set of defined tests for their breed, while others rely on genetic counseling, provided ideally from the testing laboratory, and also from breeder colleagues. Direct-to-consumer testing requires the utmost transparency and clear communication with the customer. Knowledgeable veterinary and genetics professionals should be involved to make sure that the appropriate tests are being ordered, to provide the interpretation of the results, and to deliver genetic counseling if needed. The consumer needs to know if the results provided are actionable and the laboratory should provide the scientific evidence on which the test is based (ACMG Statement on direct-to-consumer genetic testing 2008). The standards and guidelines provide for minimal and desired guidelines for personnel and content that should be included on the clinical laboratory websites to communicate important aspects of the particular test including frequency of the mutation in the breed, if known, the penetrance and expressivity of the condition, and the appropriate breeds for particular mutations. In addition, companies should provide genetic counseling on all traits and diseases for which they offer testing. As discussed below, non-peer reviewed mutations should be labeled as investigational, with clarity to the consumer that they have not been independently validated outside of the offering institution. The identification of the mutation in a research laboratory does not constitute an independent clinical or laboratory validation.

## Genetic screening versus diagnostic testing

Genetic screening approaches have been used in human genetic testing in many ways such as to identify inborn errors of metabolism in newborns (e.g., newborn screening, Weismiller 2017), preconception carrier screening (Grody et al. 2013) or to identify possible cytogenetic aneuploidies in noninvasive prenatal testing (e.g., NIPT, Cherry et al. 2017). Abnormalities identified by these genetic screens are followed up by confirmatory testing prior to making any irreversible decision. A genetic screen provides a single piece of evidence for a particular genotype of an individual; whereas diagnostic testing provides two or more pieces of evidence supporting that the genotype provided accurately represents the true genotype of that individual allowing actionable treatments and sometimes irreversible decisions. Distinguishing the difference between a genetic screen and a diagnostic test is important to clinical canine testing so that veterinarians and consumers can understand which results can be acted upon from those results that may require follow-up or confirmation.

With the completion of the sequencing of the canine genome (Lindblad-Toh et al. 2005), multiplex analysis of hundreds or thousands of genomic targets is now possible. Canine arrays are available from at least two commercial providers (Illumina, San Diego, CA, USA; Affymetrix, Thermo Fisher Scientific, Waltham, MA, USA) and provide the ability to screen a dog’s genome for more than 100,000 targets. A few laboratories are providing clinical services to the consumer using these tools with no guidance on which diseases or mutations to include on these genetic screens. Even if the microarray or method provides for more than one probe to a particular target, because the probes are interrogated on a single device and performed at the same time, subjected to the same methods and conditions, these platforms do not meet the desired standard as published (Shaffer et al. 2018), nor do they meet the requirements of methods-based proficiency testing (Schrijver et al. 2014). To comply with the standards, laboratories providing such services must develop a second test on a different method that can be used as an independent, confirmatory test to provide a level of diagnostic testing that can identify allele dropout or nonamplification of certain alleles (Ramirez et al. 2019) and to meet the desired standard, or if the confirmatory test is not available or not conducted, then the testing must be labeled as a genetic screen rather than a diagnostic test to conform to the standards (Shaffer et al. 2018). These direct to consumer tests should be labeled clearly as a genetic screen on the laboratories’ websites so that the veterinarian, dog owner or breeder understands that no irreversible decisions (such as spay, neuter or euthanasia) should be made on the basis of such testing.

## Rare or private mutations

One of the dilemmas that canine testing laboratories face is whether to offer an apparently private mutation to breeders in the chance that it is found in rare individuals of the breed. Researchers strive to test new mutations identified in a single family within a breed and among many breeds to distinguish a rare mutation from a private mutation. In this issue of *Human Genetics*, the paper by Murphy et al. (2019) describes the finding of a mutation in the *SLC6A5* gene, predicated to cause a frameshift and premature stop codon in a family of Spanish greyhound dogs with hyperekplexia or startle disease. The authors screened 34 unrelated greyhounds, 659 domestic dogs of pure and mixed breeds, and 54 wild canids and did not find the mutation, suggesting that it may be private to this one greyhound family. The quandary is whether clinical testing laboratories should develop and offer such a test, knowing that it cannot be fully validated, as affected or carrier dog samples may not be available. The reasons for offering such a test may include the chance of finding the mutation in other members of the breed not accessed by the original researchers, potentially identifying the mutation in other breeds through genetic screening that can be further clinically validated or being able to offer the test to veterinarians that may have a dog with clinical signs.

Offering a test for a rare or private mutation has raised concerns in the canine community (DogWellNet.com 2019), but, once again, the ACMG has faced this challenge with human testing and has produced a guideline on the use of genetic testing for ultra-rare disorders and private mutations that can provide some guidance (Maddalena et al. 2005). In evaluating whether to add a new disease test to the clinical laboratory, the published standards and guidelines (Shaffer et al. 2018) and checklist (S1, Sect. 2.1) are helpful in establishing whether a new test meets certain clinical validation criteria to strongly support that the mutation is associated with the phenotype, thus demonstrating clinical utility. If clinical utility is established or the evidence is strong for clinical utility, a seemingly private mutation can be offered to those that desire this test as long as the information provided about the test clearly states that the frequency in the breed has not been established and that the mutation may represent a private mutation restricted to a single family or breed line.

## An analysis of clinical utility of canine genetic tests

Demonstrating clinical utility, the ability for a test to reliably identify individuals who have or will develop the disorder, is of utmost importance for any new test. In the interest of ethical practice and maintenance of consumer trust, efforts should be made by clinical laboratories to accurately interpret and report the clinical utility of genetic test offerings. One example of an available genetic test with questionable clinical utility is for a mutation in the canine *GPT* gene (Chu 2017; White et al. 2015) which has been associated with a reduction in the serum activity of an enzyme called alanine aminotransferase (ALT). A publication by White et al. (2015) determined that dogs either heterozygous or homozygous for a *GPT* mutation displayed significantly lower serum ALT values than dogs without the mutation, with dogs homozygous for the variant showing greater decreases. Because increases in ALT activity are commonly recognized as a marker for hepatocellular damage in dogs, the perceived concern was that dogs with liver disease which have inherited the *GPT* mutation may not be identified on standard serum biochemistry panels. The authors claimed that affected dogs may have ALT values which still fall into the normal laboratory reference range despite being elevated for that particular individual. Despite scarce information and statistics supporting the use of this test result in a clinical setting, a test to determine the number of copies of the *GPT* mutation in dogs is commercially available to the public.

In reference to *GPT* genetic test results, the commercial offering states that: “…as a veterinarian, this is a valuable piece of information that can be used to accurately assess your dog’s health” (Chu 2017). However, graphs display findings that a subset of dogs with liver disease had ALT values within the laboratory reference range regardless of the number of mutation copies inherited, including those dogs with no copies of the mutation (White et al. 2015). In addition, dogs with ALT values falling in the normal range were not further compared statistically to determine specificity or sensitivity of the genetic test result in relation to the likelihood of an actual hepatopathy. With such limited information available, a veterinarian would find it challenging to make a sound clinical interpretation of the genetic test result. It could be argued that at present, baseline ALT values obtained through serum biochemistry of a young, healthy dog would be much more informative for a future liver disease diagnosis for the same dog than the results of a *GPT* DNA-based mutation test for which the clinical utility has not been demonstrated.

In addition to a dearth of statistics supporting the use of the *GPT* mutation in a clinical setting, further complicating the case for clinical utility is the finding that about half of the dogs tested had genotypes consistent with a low normal baseline ALT activity (Chu 2017; White et al. 2015). If assumed to be accurate that half of the canine population has low ALT activity, one would expect the ALT values of these dogs to weigh heavily in the establishment of laboratory reference values during the standard practice of performing random sampling of healthy individuals from the population (Horowitz et al. 2010). Therefore, logic suggests that dogs with the *GPT* mutation and a normal ALT value, despite the presence of liver disease, may be exceptionally rare since a random sampling would include a large number of dogs with low ALT that would influence the reference range size. In addition, it has not been determined how much more likely it is for dogs with liver disease and the *GPT* mutation to have ALT values in the normal range compared to diseased dogs that have not inherited the mutation.

Lastly, diagnosis of liver disease in the study was not made using a liver biopsy, the commonly recognized standard for diagnosing chronic hepatitis and hepatic fibrosis in dogs (Lidbury 2017). Instead, diagnosis was based on an increase in serum aspartate aminotransferase (AST) activity, an enzyme found primarily in liver and muscle tissues. Though investigators took precautions to exclude dogs with myopathy-induced increases in AST, given the clinical diagnostic superiority of liver histopathology over biochemistry profiles in hepatopathies, it could be argued that the former would be most relevant to assess the clinical utility of the *GPT* mutation test.

As published in this issue of *Human Genetics*, Pindar and Ramirez (2019) examined ALT values in dogs with an at-risk genotype for copper toxicosis. Mutations in *ATP7B* have been associated with copper toxicosis in Labrador Retrievers, while a mutation in *ATP7A* provides some protection against disease development (Fieten et al. 2016). Pindar and Ramirez (2019) examined the genotypes in 40 Labrador Retrievers and Labrador Retriever crosses located in a single service dog facility. Serum ALT levels, hepatic copper concentrations, and hepatic histopathology were examined in ten offspring from parents who were both heterozygous for the *ATP7B* mutation. Five offspring were homozygous mutant, four were heterozygous, and one was homozygous normal. None of the dogs had increased serum ALT activity, but all dogs homozygous for the *ATP7B* mutation had elevated hepatic copper concentrations after liver biopsy regardless of sex or presence of the protective *ATP7A* mutation. Thus, in this study, serum ALT activity levels were not helpful in identifying early signs of liver disease. Although the *GTP* mutation was not examined in this study, it is not clear how knowing the *GTP* genotype would be helpful in the absence of a liver biopsy in dogs at risk for copper toxicosis.

In this particular example, while the original finding of mutations in *ATP7B* and *ATP7A* was not surprising because mutations in these orthologous genes have been found in defects in copper metabolism in humans (OMIM #277900 Wilson Disease and OMIM #309400 Menkes Disease, respectively) (Fieten et al. 2016), further research in conjunction with a clinical testing facility able to provide genotyping was necessary to better understand the implications of at-risk genotypes in the Labrador Retriever (Pindar and Ramirez 2019).

## Identification of a breed-specific mutation in other, non-related breeds

Whether a mutation is found in a single family, a single breed or across multiple breeds provides a clue as to when the mutation may have arisen. Mutations in a single family represent private mutations that can become widespread within a breed, especially when a particular dog with the mutation becomes a ‘popular sire’ in which the dog is used disproportionately for breeding within a breed. These mutations may be restricted to a breed if there is no outcrossing with other purebred dogs. However, many mutations are found across various, thought to be dissimilar breeds, suggesting that a mutation arose prior to breed development. An example of a widespread mutation is the mutation in the *SOD1* gene associated with canine degenerative myelopathy (DM) (Zeng et al. 2014). Although the mutation is found in about 125 breeds, there is limited evidence that this mutation causes clinical symptoms of DM in all homozygous individuals across different breeds. Zeng et al. (2014) were able to evaluate the spinal cord histopathology in at least one individual in about 23 dog breeds, but that leaves about 100 breeds for which the spinal cords have not been examined. As an example, in our laboratory (Paw Print Genetics), we have identified the homozygous, at-risk genotype in seven healthy Norfolk Terriers. Although 5 of them are under the age of 4, two dogs are ages 10 and 12 and should have developed the disease based on the age of onset for other breeds. The lack of disease development could be due to other genetic modifiers in the genome (Ivansson et al. 2016), delayed age of onset in certain breeds, or other unknown genetic or environmental factors.

Determining whether discordant phenotypes are due to breed-specific differences or perhaps misidentification of a benign polymorphism as a causative mutation is difficult. As an example of misinterpretation of a benign polymorphism is the fairly recent report of von Willebrand disease, type 2 in a large number of breeds (Donner et al. 2018), with no evidence of a bleeding disorder except in the German shorthaired pointer (GSP) (Vos-Loohuis et al. 2017). This discrepancy was recently reconciled with the realization that the original mutation reported (Kramer et al. 2004) was a polymorphism that was in linkage disequilibrium to the causative mutation in the GSP, but not linked in the other breeds (Vos-Loohuis et al. 2017). Another example concerns subvalvular aortic stenosis (SAS) in the Newfoundland dog (Stern et al. 2014). We and others (Drögemüller et al. 2015) identified that the purported disease-causing mutation was present in many breeds other than Newfoundlands with no clinical signs of SAS. The identification of the mutation in many breeds that had no history of the disease led us to quickly pull this test from our laboratory (Paw Print Genetics, unpublished data) and left us explaining to customers why the test was pulled and why caution should be used when interpreting any findings reported from other laboratories for this variant. Although researchers should test as many dogs within and across many breeds as possible when a new mutation has been identified, it is the clinical laboratories that likely have access to samples from a large number of breeds and can better assess the clinical utility of the test.

In addition, the use of a single SNP array design across all breeds will lead to the identification of mutations in new breeds, not originally found during the course of research (Donner et al. 2018). However, how that information is delivered to the consumer is the responsibility of the clinical laboratory. For example, a mutation in *PDK4* found in dilated cardiomyopathy (DCM) in the Doberman Pinscher (Meurs et al. 2012) is also found in many other breeds, with and without history of DCM. Although in our laboratory (Paw Print Genetics), the *PDK4* mutation associated with DCM is listed only for the Doberman Pinscher; it can be ordered in other purebred and mixed breed dogs. In these cases, if the mutation is identified, we counsel the customer that we do not know if the finding of this mutation puts their dog at an increased risk over the general breed population but that they may want to follow up with a veterinary cardiologist and follow any recommended guidelines for monitoring. In a paper published in this issue of *Human Genetics*, Meurs and her colleagues have found a second mutation, this time in the *TTN* gene, that seems to contribute to this complex disease (Meurs et al. 2019). This variant was not found after screening 125 unaffected, non-Doberman Pinscher dogs from 23 different breeds. However, when a test for this mutation becomes commercially available and after screening thousands of dogs in a clinical laboratory, this mutation may be identified in other breeds. Therefore, continual monitoring of the performance of a clinical test within and across breeds is an important aspect of a laboratory’s quality assurance program.

## Offering non-peer reviewed clinical tests

In 2013, the Supreme Court of the United States unanimously ruled that naturally occurring DNA sequences cannot be patented (reviewed in: Klein 2013). This decision removed a substantial hurdle to clinical testing laboratories by allowing testing of variations in the genome associated with disease in which the genes had previously been patented. This decision promotes better patient access to testing and the ability to get a second opinion from more than one testing laboratory, if necessary. Removing patents from the equation allows for multiple laboratories to develop a clinical test, which should decrease prices and promote continued research and discovery on these diseases.

As in humans, finding mutations associated with diseases in dogs is an important contribution to understanding the genetic basis of canine inherited conditions as well, but do not constitute ‘inventions’ and therefore, because they are naturally occurring substances, cannot be patented. Unfortunately, rather than celebrating that breeders now have access to testing for these conditions, many researchers and clinical testing laboratories are not publishing new mutations, but rather are keeping their findings as trade secrets so that they are the only ones who can offer the test. Setting aside the fact that many discoveries are funded with public grants, the act of not submitting the mutations for peer review and independent confirmation is counter to the entire scientific method that is crucial for the process of validation of findings and contrary to the ethical standards for which all scientists are held. Errors and misattributed mutations are more likely to occur without peer review and these errors will erode customer confidence in the veterinary genetic testing industry, hurting dog owners and testing laboratories in the long run. As seen in human genetics prior to the Supreme Court decision, limitations in the number of laboratories allowed to perform certain testing may result in increased prices of these tests to breeders, dissuading them from testing at all. In addition, more importantly, this practice impacts access to the test by breeders and veterinarians that are forced to use a single laboratory, which may be located in another country. In addition, these tests cannot be clinically validated by other laboratories, which may lead to the offering of a test that may not be detecting a disease-causing mutation that would have been independently validated and uncovered by other laboratories. Although the standards and guidelines cannot change this new and detrimental trend, in these cases, any unpublished, non-peer reviewed mutation test that is offered to the public should be clearly labeled as investigational on the company’s website with an explanation that investigational means that it has not been independently reviewed or validated.

## Summary

The use of genetic testing in dogs is useful to the breeder who wishes to avoid producing puppies with diseases, provides tests for the veterinarian confirming a clinical diagnosis and allows selection of dogs for various important duties within our society (Shaffer et al. 2017). Without genetic testing, it is difficult for the breeder to effectively reduce the mutation in their breeding lines, as genetic testing allows the breeder to identify carrier individuals. By breeding carriers to genetically normal individuals, breeders eliminate the possibility of the disease in the puppies and help maintain diversity within the breed. It is the clinical testing laboratory’s responsibility to ensure accurate results that can be relied on to make decisions regarding breeding, neutering and even euthanasia. The field of canine genetics is undergoing a rapid explosion of new mutation identification, akin to what was observed in human genetics since the first published human genome sequence. Keeping up with the number of new disease and trait mutations being identified is a challenge for the clinical laboratory. The published standards and guidelines and the checklist available to all laboratories should assist in providing a structure by which the laboratories can begin to develop or enhance their quality assurance programs under which new tests are developed, validated and launched. Acknowledging and embracing this challenge by working together will provide higher standards in canine testing which should lead to more accurate results. This is the responsible approach to providing the consumer the best care for their dogs.

## Electronic supplementary material

Below is the link to the electronic supplementary material.

Supplementary material 1 (DOCX 48 kb)
