# Supplementary material for: Quality assurance checklist and additional considerations for canine clinical genetic testing laboratories: a follow-up to the published standards and guidelines
Source: Hum Genet. 2019 Apr 13;138(5):501–8. doi: 10.1007/s00439-019-02013-9 (PMC6536464; doi:10.1007/s00439-019-02013-9)
Supplement: Supplementary file 1 — Supplementary material 1 (DOCX 48 kb) [file 439_2019_2013_MOESM1_ESM.docx]

**Standards and Guidelines for Canine Clinical Genetic Testing Laboratories – Checklist**

Self-assessment conducted on:

Note: *Laboratories should meet the minimum standards and strive to meet the desired standards. For minimum standards not met, the laboratory should provide a plan and timeline for meeting those standards. If all minimum standards are met, reassessment should occur in one year. For those minimum standards not met, reassessment should occur in 6 months. Each year, the laboratory should strive to complete at least one new desired standard. Goals for completing the desired standards can be noted on this self-assessment. For any item marked N/A, an explanation should be added in the notes.*

**DEFINITIONS**

**Analytical sensitivity**

The proportion of samples with a known mutation that are correctly classified/identified with the at-risk genotype or trait

**Analytical specificity**

The proportion of samples with no known mutation that are correctly classified/identified with the wildtype (normal) genotype for the disease or trait

**Analytical validity**

The ability to accurately and reliably identify a mutation of interest in the sample type that will be used clinically

**Clinical specificity**

The proportion of unaffected individuals who will be identified by the test as negative (normal, homozygous wildtype)

**Clinical utility**

The ability of a clinical test to reliably identify individuals who have or will develop the disorder or trait

**LMS**

Laboratory management system

**MBP**

Methods-based proficiency

**Positive predictive value of a test**

The proportion of positive test results that correctly identify individuals with the disease or trait

**SOP**

Standard operating procedures

| **1.** | **GENERAL REQUIREMENTS** | | | | |
| --- | --- | --- | --- | --- | --- |
| **1.1** | **Personnel** | | | | |
| 1.1.1 | Minimum Standard: | Yes | No | N/A | Notes |
| 1.1.1.1 | The laboratory has a laboratory director or technical supervisor on site? | ☐ | ☐ | ☐ |  |
| 1.1.1.2 | The laboratory director and/or technical supervisor has an appropriate degree (PhD, DVM, or equivalent)? | ☐ | ☐ | ☐ |  |
| 1.1.1.3 | The laboratory director and/or technical supervisor has at least 2 years of training in a clinical genetics laboratory? | ☐ | ☐ | ☐ |  |
| 1.1.1.4 | The laboratory has a medical director on site? | ☐ | ☐ | ☐ |  |
| 1.1.1.5 | The medical director has a DVM (or equivalent)? | ☐ | ☐ | ☐ |  |
| 1.1.1.6 | The medical director has an appropriate and valid license? | ☐ | ☐ | ☐ |  |
| 1.1.1.7 | Clinical laboratory technologists/technicians have an undergraduate degree in a relevant scientific field? | ☐ | ☐ | ☐ |  |
| 1.1.1.8 | List number of technologists/technicians that fulfill this requirement: |  | | | |
|  | | | | | |
| 1.1.2 | Desired Standard: | Yes | No | N/A | Notes |
| 1.1.2.1 | The laboratory has both a laboratory/technical director and medical director on site? | ☐ | ☐ | ☐ |  |
| 1.1.2.2 | The laboratory/technical director is board certified in genetics or molecular pathology? | ☐ | ☐ | ☐ |  |
| 1.1.2.3 | The medical director or DVM laboratory director is board certified in a veterinary subspecialty? | ☐ | ☐ | ☐ |  |
| 1.1.2.4 | Clinical laboratory technologists/technicians have 5 years of relevant laboratory experience? | ☐ | ☐ | ☐ |  |
| 1.1.2.5 | List number of technologists/technicians that fulfill this requirement: |  | | | |
|  | | | | | |
| **1.2** | **Facilities** | | | | |
| 1.2.1 | Minimum Standard: | Yes | No | N/A | Notes |
| 1.2.1.1 | Laboratory space, equipment and facilities are sufficient to ensure safe, accurate and acceptable standards of performance? | ☐ | ☐ | ☐ |  |
| 1.2.1.2 | Laboratory equipment is maintained, cleaned, and monitored at appropriate intervals? | ☐ | ☐ | ☐ |  |
| 1.2.1.3 | Records of maintenance are recorded and kept? | ☐ | ☐ | ☐ |  |
| 1.2.1.4 | SOPs are in place for sample handling? | ☐ | ☐ | ☐ |  |
| 1.2.1.5 | SOPs are in place for minimizing contamination? | ☐ | ☐ | ☐ |  |
| 1.2.1.6 | Laboratory areas are designated and physically separated for reagent preparation, sample preparation, and PCR amplification/detection? | ☐ | ☐ | ☐ |  |
| 1.2.1.7 | Separate rooms are designated for pre-PCR and post-PCR workspaces? | ☐ | ☐ | ☐ |  |
| 1.2.1.8 | Pre-PCR and Post-PCR rooms have appropriate air handling with positive or negative pressures that are monitored and maintained? | ☐ | ☐ | ☐ |  |
| 1.2.1.9 | Pre-PCR and Post-PCR rooms have dedicated equipment? | ☐ | ☐ | ☐ |  |
| 1.2.1.10 | Laboratory workflow ensures unidirectional flow from pre-PCR to post-PCR areas to reduce the possibility of sample contamination? | ☐ | ☐ | ☐ |  |
|  | | | | | |
| **1.3** | **Quality Practices** | | | | |
| 1.3.1 | Minimum Standard: | Yes | No | N/A | Notes |
| 1.3.1.1 | The laboratory has a quality manual that is maintained for all laboratory processes? | ☐ | ☐ | ☐ |  |
| 1.3.1.2 | The quality manual is reviewed annually by all staff and the laboratory director and updated as needed? | ☐ | ☐ | ☐ |  |
| 1.3.1.3 | The laboratory directors and staff have reviewed and signed each SOP? | ☐ | ☐ | ☐ |  |
| 1.3.1.4 | The quality manual includes protocols and work instructions for all aspects of the testing process including assay development and validation? | ☐ | ☐ | ☐ |  |
| 1.3.1.5 | The quality manual includes protocols for specimen handling? | ☐ | ☐ | ☐ |  |
| 1.3.1.6 | The quality manual includes protocols for receipt and storage of samples? | ☐ | ☐ | ☐ |  |
| 1.3.1.7 | The quality manual includes protocols for all testing procedures? | ☐ | ☐ | ☐ |  |
| 1.3.1.8 | The quality manual includes protocols for data review and reporting? | ☐ | ☐ | ☐ |  |
| 1.3.1.9 | The laboratory has a documented quality management system that includes quality control (QC), quality assurance (QA), quality improvement (QI) and corrective and preventative action (CAPA) plans to assure that all reagents, equipment, methodologies, and personnel operate at optimum levels? | ☐ | ☐ | ☐ |  |
| 1.3.1.10 | The laboratory quality management system is reviewed annually by the laboratory and/or medical director? | ☐ | ☐ | ☐ |  |
| 1.3.1.11 | All laboratory developed in-house tests have a development plan and validation plan? | ☐ | ☐ | ☐ |  |
| 1.3.1.12 | The laboratory has an SOP in place for releasing new tests? | ☐ | ☐ | ☐ |  |
| 1.3.1.13 | The SOP for each test is reviewed annually and modified if standard practices have deviated from the plan? | ☐ | ☐ | ☐ |  |
| 1.3.1.14 | The laboratory has an SOP in place for how disputed results are resolved? | ☐ | ☐ | ☐ |  |
| 1.3.1.15 | Results of concern or disputed by the customer are investigated and repeat testing or other actions to confirm the original results are detailed in the SOP? | ☐ | ☐ | ☐ |  |
| 1.3.1.16 | The laboratory has a disputed results log that details the customer concern and how those concerns were resolved? | ☐ | ☐ | ☐ |  |
| 1.3.1.17 | The laboratory disputed results log is reviewed on a regular basis to ensure that customer concerns are resolved? | ☐ | ☐ | ☐ |  |
|  | | | | | |
| 1.3.2 | Desired Standard: | Yes | No | N/A | Notes |
| 1.3.2.1 | The laboratory has implemented a methods-based proficiency (MBP) testing protocol for each mutation region offered for clinical testing? | ☐ | ☐ | ☐ |  |
| 1.3.2.2 | The laboratories MBP includes at least two independent methods for each mutation region interrogated with non-overlapping primers, when possible depending on the genomics region, between the two assays to minimize allele dropout due to unforeseen polymorphisms in individual samples? | ☐ | ☐ | ☐ |  |
| 1.3.2.3 | For mutation regions that cannot be developed on two different methods, has the laboratory ensured that the two primer sets used in PCR do not contain significant sequence overlap? | ☐ | ☐ | ☐ |  |
| 1.3.2.4 | As part of the MBP testing, the laboratory compares the genotype results from the two assays before reporting the result? | ☐ | ☐ | ☐ |  |
| 1.3.2.5 | The laboratory has an SOP in place to deal with discordant genotypes? | ☐ | ☐ | ☐ |  |
| 1.3.2.6 | The discordant genotype SOP includes performing a third independent assay, repeating the testing on the original sample, or obtaining a new sample to resolve the discordant results? | ☐ | ☐ | ☐ |  |
| 1.3.2.7 | The laboratory has a quality committee that regularly meets to review the quality metrics being measured for the laboratory? | ☐ | ☐ | ☐ |  |
| 1.3.2.8 | The quality committee regularly reviews adverse events and non-conforming products, laboratory errors, customer complaints, quality improvement reports, and corrective or preventative actions due to issues identified by the routine quality assurance and quality control practices? | ☐ | ☐ | ☐ |  |
| 1.3.2.9 | The quality committee ensures that laboratory staff are properly trained? | ☐ | ☐ | ☐ |  |
| 1.3.2.10 | The laboratory documents staff training as part of the overall quality system? | ☐ | ☐ | ☐ |  |
| 1.3.2.11 | The quality committee performs routine internal audits of the laboratory to ensure that the established quality system is being followed? | ☐ | ☐ | ☐ |  |

| **1.4** | **Privacy** | | | | |
| --- | --- | --- | --- | --- | --- |
| 1.4.1 | Minimum standard: | Yes | No | N/A | Notes |
| 1.4.1.1 | The laboratory has a privacy policy that is maintained and reviewed annually? | ☐ | ☐ | ☐ |  |
| 1.4.1.2 | Records are maintained in a manner that ensures privacy for the dog owner? | ☐ | ☐ | ☐ |  |
| 1.4.1.3 | Laboratory SOPs regarding privacy includes that records are only accessible by designated laboratory staff that have been instructed about the laboratory’s privacy policy? | ☐ | ☐ | ☐ |  |
| 1.4.1.4 | Laboratory SOPs regarding privacy includes that records are released only to dog owners/breeders or veterinarians who submitted the samples and/or placed the order on the dog unless instructed otherwise by the owner/breeder or veterinarian? | ☐ | ☐ | ☐ |  |
|  | | | | | |
| 1.4.2 | Desired Standard: | Yes | No | N/A | Notes |
| 1.4.2.1 | Laboratory SOPs regarding privacy includes that results are released to third parties only after proper written authorization by the owner, breeder, or veterinarian? | ☐ | ☐ | ☐ |  |
|  | | | | | |
| **2.** | **TEST VALIDATION** | | | | |
| **2.1** | **Clinical Validation** | | | | |
| 2.1.1 | Minimum Standard: | Yes | No | N/A | Notes |
| 2.1.1.1 | For each clinical test offered, the laboratory has critically reviewed of one or more peer-reviewed publication that provides strong evidence that a particular gene mutation causes or is strongly associated with a clinical disease or phenotypic trait? | ☐ | ☐ | ☐ |  |
| 2.1.1.2 | For each clinical test offered, the laboratory has evaluated whether there is significant co-segregation of the mutation with the disease or trait? | ☐ | ☐ | ☐ |  |
| 2.1.1.3 | For each clinical test offered, the laboratory has evaluated whether the mutation was identified in a single affected individual, single pedigree, found widely within the breed or across many breeds? | ☐ | ☐ | ☐ |  |
| 2.1.1.4 | For each clinical test offered, the laboratory has evaluated whether functional studies of the mutation were performed? | ☐ | ☐ | ☐ |  |
| 2.1.1.5 | For each clinical test offered, the laboratory has evaluated whether the test will provide relevant information for diagnosis, prognosis, treatments, breeding decisions, or veterinary surveillance? | ☐ | ☐ | ☐ |  |
| 2.1.1.6 | The laboratory has determined for each clinical test offered that the data in the publication are sufficient to determine the sensitivity and specificity of the test being developed? | ☐ | ☐ | ☐ |  |
| 2.1.1.7 | The laboratory has determined for each clinical test offered that the data are sufficient to determine the clinical utility of the test? | ☐ | ☐ | ☐ |  |
|  | | | | | |
| 2.1.2 | Desired Standard: | Yes | No | N/A | Notes |
| 2.1.2.1 | Test for which the clinical utility is not clear from the publications, the laboratory has worked with breed clubs and breeders to understand their desire to establish a clinical test for the published mutation? | ☐ | ☐ | ☐ |  |
| 2.1.2.2 | Test for which the clinical utility is not clear from the publications, the laboratory has collected samples from normal dogs and from those with the disease or displaying the trait for in-house analytical validation? | ☐ | ☐ | ☐ |  |
| 2.1.2.3 | The laboratory has established the clinical specificity of each test? | ☐ | ☐ | ☐ |  |
| 2.1.2.4 | The laboratory has established the positive predictive value of each test? | ☐ | ☐ | ☐ |  |

| **2.2** | **Analytical Validation** | | | | |
| --- | --- | --- | --- | --- | --- |
| 2.2.1 | Minimum Standard: | Yes | No | N/A | Notes |
| 2.2.1.1 | The analytical validity of each test in the sample type that will be used clinically has been determined? | ☐ | ☐ | ☐ |  |
| 2.2.1.2 | The laboratory has validated all in-house assays for each mutation regardless of what is stated in a peer-reviewed publication? | ☐ | ☐ | ☐ |  |
| 2.2.1.3 | For any specific mutation, the assay has been validated on a number of sample types (cheek swabs, blood, semen, etc.) with known genotypes? | ☐ | ☐ | ☐ |  |
| 2.2.1.4 | For some assays in which the mutant genotype may be rare and unavailable to assess the analytical sensitivity, the laboratory is offering the new test after validation with normal (wildtype) samples while it continues to collect the test results and correlate those with clinical diagnosis and pedigree history? | ☐ | ☐ | ☐ |  |
| 2.2.1.5 | For each test, the limitations have been determined, and any variables identified and monitored for continued high-level performance of the test? | ☐ | ☐ | ☐ |  |
| 2.2.1.6 | For each test, primers and probe sequences were subjected to a BLAST search to identify homologous regions in the genome that might be targets and interfere with the accuracy of the test and those identified have been documented? | ☐ | ☐ | ☐ |  |
| 2.2.1.7 | For each test, the genomic regions of interest have been examined using the public literature and databases to identify known variation that might interfere with primer binding sites during primer design? | ☐ | ☐ | ☐ |  |
| 2.2.1.8 | For each test, primers, probes, PCR conditions, expected wildtype and mutation sizes of amplicons, map positions noting autosomal or X-linked, and other specifications regarding the methodologies used for each test has been documented? | ☐ | ☐ | ☐ |  |
| 2.2.1.9 | For each test, primers, probes, PCR conditions, expected wildtype and mutation sizes of amplicons, map positions noting autosomal or X-linked, and other specifications regarding the methodologies used for each test is reviewed on an ongoing basis? | ☐ | ☐ | ☐ |  |
|  | | | | | |
| 2.2.2 | Desired Standard: | Yes | No | N/A | Notes |
| 2.2.2.1 | The laboratory has obtained samples from other laboratories, breed clubs and breeders for which the genotypes and/or clinical diagnoses are known for validation of each assay? | ☐ | ☐ | ☐ |  |
| 2.2.2.2 | The laboratory has developed two independent assays for each mutation region as part of their internal MBP testing program? | ☐ | ☐ | ☐ |  |
| 2.2.2.3 | The laboratory tracks and periodically reviews the performance of any confirmatory or duplicate testing performed? | ☐ | ☐ | ☐ |  |
| 2.2.2.4 | Each assay is evaluated for reproducibility as part of the performance standards of each test? | ☐ | ☐ | ☐ |  |
| 2.2.2.5 | The laboratory has an on-going collection of patient outcomes for continual monitoring of the clinical validity of a test? | ☐ | ☐ | ☐ |  |

| **3.** | **PREANALYTICAL STANDARDS** | | | | |
| --- | --- | --- | --- | --- | --- |
| **3.1** | **Customer Education and Websites** | | | | |
| 3.1.1 | Minimum Standard: | Yes | No | N/A | Notes |
| 3.1.1.1 | The laboratory website includes a detailed description of the available disease tests? | ☐ | ☐ | ☐ |  |
| 3.1.1.2 | The laboratory website includes a detailed description of appropriate breeds for each test? | ☐ | ☐ | ☐ |  |
| 3.1.1.3 | The laboratory website includes a detailed description of the clinical signs of the condition? | ☐ | ☐ | ☐ |  |
| 3.1.1.4 | The laboratory website includes the mode of inheritance for each condition? | ☐ | ☐ | ☐ |  |
| 3.1.1.5 | The laboratory website includes a detailed description of appropriate uses and limitations of each test? | ☐ | ☐ | ☐ |  |
| 3.1.1.6 | The laboratory website includes turnaround times and pricing for each test? | ☐ | ☐ | ☐ |  |
| 3.1.1.7 | When available, the laboratory website includes a detailed description of disease variability, prevalence, penetrance, age of onset, treatment options, and life expectancy for dogs with the condition? | ☐ | ☐ | ☐ |  |
| 3.1.1.8 | The laboratory website includes the gene and mutation for each test? | ☐ | ☐ | ☐ |  |
| 3.1.1.9 | The laboratory website identifies any nonpublished, nonpeer-reviewed mutations as investigational? | ☐ | ☐ | ☐ |  |
| 3.1.1.10 | The laboratory/technical director and/or medical director are available to answer any questions that owners, breeders or veterinarians might have regarding the disease and appropriate uses of the test? | ☐ | ☐ | ☐ |  |
| 3.1.1.11 | The laboratory website includes a detailed description on acceptable sample types, how to obtain, store and ship the sample, and sample quantity to submit to the laboratory? | ☐ | ☐ | ☐ |  |
|  | | | | | |
| 3.1.2 | Desired Standard: | Yes | No | N/A | Notes |
| 3.1.2.1 | The laboratory website displays the most commonly used names, abbreviations and acronyms for each disease, condition or trait in a way that minimizes confusion that may lead to ordering the wrong test? | ☐ | ☐ | ☐ |  |
| 3.1.2.2 | The laboratory works with other testing laboratories to achieve uniformity for disease names, abbreviations, and acronyms across all testing laboratories? | ☐ | ☐ | ☐ |  |
| 3.1.2.3 | The laboratory website includes any differential diagnoses that could be considered, the clinical sensitivity, and positive predictive values for each test when available? | ☐ | ☐ | ☐ |  |
| 3.1.2.4 | For certain diseases in which no causative mutation has been identified and linked markers or risk alleles are reported, are these tests clearly labeled on the website as ‘associated markers’ to distinguish them from known causative gene mutations? | ☐ | ☐ | ☐ |  |
| 3.1.2.5 | For any associated markers, the website clearly describes the clinical utility? | ☐ | ☐ | ☐ |  |
|  | | | | | |
| **3.2** | **Pretest Counseling** | | | | |
| 3.2.1 | Minimum Standard: | Yes | No | N/A | Notes |
| 3.2.1.1 | The laboratory has qualified individuals available to assist breeders, owners, and veterinarians with ordering the appropriate tests? | ☐ | ☐ | ☐ |  |
| 3.2.1.2 | These qualified individuals have detailed knowledge about which mutations are present in any given breed? | ☐ | ☐ | ☐ |  |
| 3.2.1.3 | The laboratory/technical director or medical director are available to answer questions about clinical validity and sensitivity of any given test? | ☐ | ☐ | ☐ |  |
| 3.2.1.4 | The laboratory employs other appropriately trained personnel such as genetic counselors that can assist customers with questions about appropriate tests to order? | ☐ | ☐ | ☐ |  |
|  | | | | | |
| **3.3** | **Specimens** | | | | |
| 3.3.1 | Minimum Standard: | Yes | No | N/A | Notes |
| 3.3.1.1 | The laboratory has an LMS for managing orders, samples, and results? | ☐ | ☐ | ☐ |  |
| 3.3.1.2 | The laboratory has SOPs detailing the laboratory standards for acceptance or rejection of specimens? | ☐ | ☐ | ☐ |  |
| 3.3.1.3 | The SOP includes optimal and acceptable specimen types? | ☐ | ☐ | ☐ |  |
| 3.3.1.4 | The SOP includes variables that affect acceptability including insufficient quantity, exposure to extreme temperatures and inappropriate collection modes? | ☐ | ☐ | ☐ |  |
| 3.3.1.5 | The laboratory ensures that customers are instructed about sample acquisition such that samples arrive in the laboratory labeled with two identifiers, which may include the dog’s name, microchip number, permanent tattoo, date of birth, laboratory number or order number? | ☐ | ☐ | ☐ |  |
| 3.3.1.6 | The laboratory ensures that samples and orders include the breed (if known), sex, and pertinent family history including related dogs that have been tested by that laboratory? | ☐ | ☐ | ☐ |  |
| 3.3.1.7 | The laboratory has a nonconforming SOP for handling samples that lack sufficient information or unique identifiers? | ☐ | ☐ | ☐ |  |
| 3.3.1.8 | The nonconforming sample SOP includes steps to contact the ordering owner, breeder, or veterinarian for the missing information? | ☐ | ☐ | ☐ |  |
| 3.3.1.9 | The nonconforming sample SOP includes on how to document and resolve the nonconforming missing information once obtained? | ☐ | ☐ | ☐ |  |
| 3.3.1.10 | The laboratory ensures that samples are accompanied by a requisition form that indicates the tests desired and identifies the ordering individual with contact information in case there are questions? | ☐ | ☐ | ☐ |  |
| 3.3.1.11 | The laboratory makes a reasonable effort to review orders and tests for appropriateness based on the breed being tested? | ☐ | ☐ | ☐ |  |
|  | | | | | |
| 3.3.2 | Desired Standard: | Yes | No | N/A | Notes |
| 3.3.2.1 | All orders are reviewed for appropriateness of the requested tests based on the breed being tested? | ☐ | ☐ | ☐ |  |
| 3.3.2.2 | The laboratory contacts the ordering individual when the sample is received by the laboratory? | ☐ | ☐ | ☐ |  |
| 3.3.2.3 | The date and time are stamped on the requisition form and recorded in LMS as received? | ☐ | ☐ | ☐ |  |
| 3.3.2.4 | The laboratory has SOPs detailing how missing information or missing unique identifiers are recorded and noted on the final laboratory report? | ☐ | ☐ | ☐ |  |
| 3.3.2.5 | The laboratory records any missing information or missing unique identifiers on the final laboratory report? | ☐ | ☐ | ☐ |  |
|  | | | | | |
| **4.** | **ANALYTICAL STANDARDS** | | | | |
| **4.1** | **Controls** | | | | |
| 4.1.1 | Minimum Standard: | Yes | No | N/A | Notes |
| 4.1.1.1 | The laboratory includes a no template in each assay to detect contamination? | ☐ | ☐ | ☐ |  |
| 4.1.1.2 | Negative controls (homozygous wildtype) are included each time an assay is performed? | ☐ | ☐ | ☐ |  |
| 4.1.1.3 | Representative positive controls for each method are run periodically for quality assurance? | ☐ | ☐ | ☐ |  |
| 4.1.1.4 | Efforts are made to include positive controls in assay runs as they become identified in the laboratory? | ☐ | ☐ | ☐ |  |
|  | |  |  |  |  |
| 4.1.2 | Desired Standard: | Yes | No | N/A | Notes |
| 4.1.2.1 | Residual clinical samples demonstrating the mutation are retained as positive controls for ongoing quality assurance and future test development? | ☐ | ☐ | ☐ |  |

| **4.2** | **Sample Preparation** | | | | |
| --- | --- | --- | --- | --- | --- |
| 4.2.1 | Minimum Standard: | Yes | No | N/A | Notes |
| 4.2.1.1 | Laboratory SOPs are in place detailing specimen handling? | ☐ | ☐ | ☐ |  |
| 4.2.1.2 | The laboratory ensures that specimens are handled and processed one at a time to prevent contamination, tampering or sample mix-up? | ☐ | ☐ | ☐ |  |
| 4.2.1.3 | Laboratory SOPs are in place detailing evaluation of sample quality at the time of sample receipt by the laboratory? | ☐ | ☐ | ☐ |  |
| 4.2.1.4 | The sample type (blood, cheek swab, tissue, semen, etc.) is recorded in the LMS noting amount and gross quality of each sample? | ☐ | ☐ | ☐ |  |
| 4.2.1.5 | Laboratory SOPs detail steps to take for compromised samples? | ☐ | ☐ | ☐ |  |
| 4.2.1.6 | The laboratory SOP for compromised samples includes contacting the submitting owner, breeder or veterinarian when a specimen does not meet laboratory requirements or if the sample appears compromised in any way (i.e. visible mold, mildew, bacterial growth, dirt, food particles for cheek swabs, clotted or lysed blood for blood samples)? | ☐ | ☐ | ☐ |  |
| 4.2.1.7 | A laboratory SOP is in place for handling unacceptable specimens? | ☐ | ☐ | ☐ |  |
| 4.2.1.8 | The laboratory has a sample retention policy regarding the original sample and extracted DNA? | ☐ | ☐ | ☐ |  |
| 4.2.1.9 | The laboratory retains any remaining original sample at least until all testing is completed and the report has been signed out? | ☐ | ☐ | ☐ |  |
|  |  | | | | |
| 4.2.2 | Desired Standard: | Yes | No | N/A | Notes |
| 4.2.2.1 | The laboratory retains any remaining original sample for six months? | ☐ | ☐ | ☐ |  |
| 4.2.2.2 | The laboratory retains the extracted DNA for three years? | ☐ | ☐ | ☐ |  |
|  |  | | | | |
| **4.3** | **Validation of Methods** | | | | |
| 4.3.1 | Minimum Standard: | Yes | No | N/A | Notes |
| 4.3.1.1 | The laboratory performs validation to demonstrate that the assay is detecting the appropriate mutation and wildtype sequence regardless of whether the assay is developed using an in-house method or purchased as a kit? | ☐ | ☐ | ☐ |  |
| 4.3.1.2 | The laboratory ensures that each assay developed and specifically designed to detect a certain mutation associated with a disease or trait is validated and its analytical specificity and analytical sensitivity are determined? | ☐ | ☐ | ☐ |  |
| 4.3.1.3 | The laboratory ensures that all multiplex assays are validated and demonstrate that all mutation regions are successfully amplified, and expected results are obtained before the test is offered? | ☐ | ☐ | ☐ |  |

| **5.** | **POSTANALYTICAL STANDARDS** | | | | |
| --- | --- | --- | --- | --- | --- |
| **5.1** | **Reporting** | | | | |
| 5.1.1 | Minimum Standard: | Yes | No | N/A | Notes |
| 5.1.1.1 | The Laboratory reports includes owner/breeder/vet and dog information: | | | | |
| 5.1.1.1.a | Order date? | ☐ | ☐ | ☐ |  |
| 5.1.1.1.b | Order number? | ☐ | ☐ | ☐ |  |
| 5.1.1.1.c | Sample receipt date? | ☐ | ☐ | ☐ |  |
| 5.1.1.1.d | Date reported? | ☐ | ☐ | ☐ |  |
| 5.1.1.1.e | Name of the person who ordered the test and/or the owner, breeder and/or veterinarian’s name? | ☐ | ☐ | ☐ |  |
| 5.1.1.1.f | Unique laboratory number of the sample? | ☐ | ☐ | ☐ |  |
| 5.1.1.1.g | Dog’s call name? | ☐ | ☐ | ☐ |  |
| 5.1.1.1.h | Breed (if known)? | ☐ | ☐ | ☐ |  |
| 5.1.1.1.i | Sex and date of birth? | ☐ | ☐ | ☐ |  |
| 5.1.1.2 | The laboratory report includes information about the testing performed: | | | | |
| 5.1.1.2.a | Disease name? | ☐ | ☐ | ☐ |  |
| 5.1.1.2.b | Gene tested? | ☐ | ☐ | ☐ |  |
| 5.1.1.2.c | Specific mutation interrogated? | ☐ | ☐ | ☐ |  |
| 5.1.1.2.d | Results obtained after genotyping? | ☐ | ☐ | ☐ |  |
| 5.1.1.2.e | Interpretation of the data? | ☐ | ☐ | ☐ |  |
| 5.1.1.2.f | Recommendations or follow-up testing required? | ☐ | ☐ | ☐ |  |
| 5.1.1.3 | Reports are signed by the laboratory/technical director and/or medical director? | ☐ | ☐ | ☐ |  |
| 5.1.1.4 | Test limitations are stated on the reports? | ☐ | ☐ | ☐ |  |
| 5.1.1.5 | Reports are issued on letterhead with the contact information for the laboratory provided? | ☐ | ☐ | ☐ |  |
| 5.1.1.6 | Unpublished, nonpeer-reviewed mutations are identified on the report as investigational? | ☐ | ☐ | ☐ |  |
|  |  | | | | |
| 5.1.2 | Desired Standard: | Yes | No | N/A | Notes |
| 5.1.2.1 | The final laboratory reports include notes regarding any verbal results to the customer, amended reports, corrected reports or notes regarding any deviation from the laboratory’s established standard practices? | ☐ | ☐ | ☐ |  |
| 5.1.2.2 | The laboratory report indicates tests that were sent to another laboratory for testing with the send-out laboratory’s contact information provided? | ☐ | ☐ | ☐ |  |
| 5.1.2.3 | The laboratory ensures that both the laboratory/technical director and medical director review results and sign out reports? | ☐ | ☐ | ☐ |  |
| 5.1.2.4 | The laboratory works with other laboratories to define acceptable nomenclature for the genotype results on reports to standardized across the industry? | ☐ | ☐ | ☐ |  |
|  |  | | | | |
| **5.2** | **Interpretations and Disclaimers** | | | | |
| 5.2.1 | Minimum Standard: | Yes | No | N/A | Notes |
| 5.2.1.1 | The laboratory website and the report have a clear and concise disclaimer? | ☐ | ☐ | ☐ |  |
| 5.2.1.2 | The disclaimer states that normal results do not exclude any undetected mutation that may be present in the gene that was tested or any other gene that was not tested? | ☐ | ☐ | ☐ |  |
| 5.2.1.3 | The disclaimer states that the presence of unexpected variation at a primer binding site could lead to allele dropout or preferential amplification of the other allele? | ☐ | ☐ | ☐ |  |
| 5.2.1.4 | The disclaimer states that the presence of mosaicism may not be detected with certain assays? | ☐ | ☐ | ☐ |  |
| 5.2.1.5 | The disclaimer states that nonpaternity may lead to unexpected results? | ☐ | ☐ | ☐ |  |
| 5.2.1.6 | The laboratory ensures that any primers found to yield nonconforming results due to variation at a primer site are to be removed from use and testing protocols? | ☐ | ☐ | ☐ |  |
|  | | | | | |
| 5.2.2 | Desired Standard: | Yes | No | N/A | Notes |
| 5.2.2.1 | The laboratory website and report show the clinical sensitivity and clinical specificity for each test? | ☐ | ☐ | ☐ |  |
|  |  | | | | |
| **5.3** | **Retention of Records and Security** | | | | |
| 5.3.1 | Minimum Standard: | Yes | No | N/A | Notes |
| 5.3.1.1 | The laboratory has a record retention policy? | ☐ | ☐ | ☐ |  |
| 5.3.1.2 | The laboratory ensures that all records are maintained in a manner that will ensure privacy, security, integrity and access? | ☐ | ☐ | ☐ |  |
| 5.3.1.3 | The laboratory computer system has been validated for security and performance including appropriate hardware, software, and back-up systems to allow uninterrupted functioning of the laboratory and prevention of data loss or security breaches? | ☐ | ☐ | ☐ |  |
|  |  | | | | |
| 5.3.2 | Desired Standard: | Yes | No | N/A | Notes |
| 5.3.2.1 | The laboratory record retention policy ensures record retention and access is maintained for a period of at least 20 years? | ☐ | ☐ | ☐ |  |

| This checklist was completed by: | |
| --- | --- |
| Title: | Date: |
|  | |
| The completed checklist was reviewed by: | |
| Laboratory/Technical Director: | Date: |
| Medical Director: | Date: |
|  | |
| Next review is to be completed on or before: | |
